# Clonal Dissemination of NDM-Producing *Proteus mirabilis* in a Teaching Hospital in Sousse, Tunisia

**DOI:** 10.3390/pathogens14030298

**Published:** 2025-03-20

**Authors:** Nadia Jaidane, Lamia Tilouche, Saoussen Oueslati, Delphine Girlich, Sana Azaiez, Aymeric Jacquemin, Laurent Dortet, Walid Naija, Abdelhalim Trabelsi, Thierry Naas, Wejdene Mansour, Rémy A. Bonnin

**Affiliations:** 1 Laboratory of Metabolic Biophysics and Applied Pharmacology (LR12ES02), Department of Biophysics, Faculty of Medicine Ibn El Jazzar of Sousse, University of Sousse, Sousse 4000, Tunisia; nadia_jaidane@famso.u-sousse.tn (N.J.);; 2 Clinical Microbiology Laboratory, University Hospital of Sahloul, Sousse 4002, Tunisia; 3 Team ‘Resist’, UMR1184 ‘Immunology of Viral, Auto-Immune, Hematological and Bacterial Diseases (IMVA-HB)’, INSERM, Université Paris-Saclay, CEA, LabEx LERMIT, Faculty of Medicine, 94270 Le Kremlin-Bicêtre, France; saoussen.oueslati@aphp.fr (S.O.); delphine.girlich@universite-paris-saclay.fr (D.G.); laurent.dortet@aphp.fr (L.D.);; 4 Faculty of Pharmacy, University of Monastir, Monastir 5000, Tunisia; 5 Faculty of Medicine Ibn El Jazzar, University of Sousse, Sousse 4000, Tunisia; 6 French National Reference Center for Antibiotic Resistance: Carbapenemase-Producing Enterobacterales, 94270 Le Kremlin-Bicêtre, France; 7 Department of Bacteriology-Hygiene, Bicêtre Hospital, APHP Paris-Saclay, 94270 Le Kremlin-Bicêtre, France; 8 Department of Anesthesia and Intensive Care, University Hospital Sahloul, Sousse 4002, Tunisia

**Keywords:** NDM-1, carbapenemase, beta-lactamase, phylogeny, VEB-6

## Abstract

*Proteus mirabilis* (*P. mirabilis*) is an opportunistic pathogen involved in urinary tract infections as well as various nosocomial infections. Emerging resistances to beta-lactams in this species complicates potential treatment since it is intrinsically resistant to colistin. Eleven isolates of carbapenem-non-susceptible *P. mirabilis* were identified in Sousse Hospital, Tunisia, from January 2018 to December 2022. MICs were determined and isolates were sequenced to determine their resistomes, sequence types, virulence factors, and their clonal relationships. Susceptibility testing showed that all isolates were resistant to carbapenems, aminoglycosides, fluoroquinolones, chloramphenicol, and the trimethoprim/sulfamethoxazole combination. They remained susceptible to the aztreonam/avibactam combination. All isolates produced NDM-1 carbapenemase and ArmA 16S rRNA methylase. In addition, one isolate co-produced the *bla*_VEB-6_ gene. All isolates belonged to ST135, and phylogenetic analysis revealed that they were closely related. This study described the first outbreak of NDM-1-producing *P. mirabilis* in Tunisia.

## 1. Introduction

Antimicrobial resistance (AMR) is a growing global public health crisis, significantly impacting patient outcomes, healthcare costs, and the effectiveness of current therapeutic strategies [1,2]. The increasing prevalence of AMR has led to a surge in difficult-to-treat infections, particularly in hospitalized patients, where multidrug-resistant (MDR) pathogens contribute to prolonged hospital stays, higher morbidity and mortality rates, and limited treatment options [3,4]. Among these pathogens, *Proteus mirabilis*, a Gram-negative rod-shaped bacterium, has emerged as a significant cause of nosocomial infections, posing serious therapeutic challenges due to its intrinsic and acquired resistance mechanisms [5].

*P. mirabilis* is notorious for its ability to swarm across agar surfaces in a characteristic bull’s-eye pattern and its urease activity, which raises urinary pH, facilitating the formation of urinary stones [6]. It is a leading cause of urinary tract infections (UTIs) and catheter-associated urinary tract infections (CAUTIs), where it rapidly colonizes catheter surfaces, leading to encrustation and increased risk of renal damage [7,8,9,10,11]. Beyond UTIs, *P. mirabilis* is an opportunistic pathogen responsible for a wide range of healthcare-associated infections, including respiratory and wound infections, peritonitis, bacteremia, meningoencephalitis, osteomyelitis, and burn infections [5,12,13,14].

The treatment of *P. mirabilis* infections is increasingly complicated due to its intrinsic resistance to tigecycline and colistin, as well as reduced susceptibility to imipenem [15]. Additionally, the bacterium frequently acquires resistance mechanisms such as extended-spectrum β-lactamases (ESBLs) and carbapenemases from Ambler class A (KPC), class B (VIM, NDM), and class D (OXA-48-like, OXA-23) [16,17,18,19,20,21,22]. Among these, the emergence of New Delhi metallo-β-lactamase (NDM)-producing *P. mirabilis* is particularly concerning, as NDM enzymes confer resistance to nearly all β-lactams, including carbapenems, either alone or in combination with β-lactamase inhibitors such as vaborbactam and relebactam [23]. This significantly limits available treatment options and contributes to the global AMR crisis.

Besides resistance, *P. mirabilis* expresses several virulence factors (VFs) associated with its pathogenicity in humans, including biofilm formation, the production of enzymes and cytotoxins, uroepithelial cell adhesion, motility, and iron acquisition systems. Additionally, this bacterium employs a phosphate transport (Pst) system and diverse iron acquisition mechanisms, including proteobactin (Pbt) and nonribosomal peptide synthetase (NRPS)-derived siderophores such as Yersiniabactin. Moreover, it secretes the hemolysins HpmA and HlyA, which act as potent toxins that disrupt host cell integrity [24,25,26].

Given the clinical importance of carbapenemase-producing *P. mirabilis*, here we report the first clonal dissemination of NDM-producing *P. mirabilis* in a university hospital in Tunisia. This study investigates the resistome, and clinical characteristics of *P. mirabilis* isolates from nosocomial infections, highlighting the urgent need for enhanced surveillance and antimicrobial stewardship to mitigate its spread.

## 2. Materials and Methods

### 2.1. Study Design and Clinical Isolates

We conducted a retrospective analysis using the Laboratory Information System to review all clinical isolates of *Proteus mirabilis* collected from various patient samples and hospital wards at the University Hospital of Sahloul (Sousse, Tunisia) between January 2018 and December 2022.

Strains exhibiting resistance to all tested antibiotics (extensively drug-resistant *Proteus mirabilis* (XDR*Pm*)), as determined by the Vitek-2 System (bioMérieux, Marcy-l’Étoile, France), were further characterized. The routinely tested antibiotics included cephalexin (CFX), ampicillin (AM), amoxicillin/clavulanic acid (AMC), ticarcillin (TIC), piperacillin (PIP), piperacillin/tazobactam (TZP), cefepime (CF), cefoxitin (FOX), cefotaxime (CTX), ceftazidime (CAZ), imipenem (IMP), ertapenem (ERT), meropenem (MP), aztreonam (ATM), amikacin (AN), gentamycin (GM), tobramycin (TM), ciprofloxacin (CIP), levofloxacine (LVX), chloramphenicol (C), tigecycline (TIG), and trimethoprim-sulfamethoxazole (SXT).

For the selected XDR*Pm* strains, clinical data were retrieved from patient medical records, while information on antibiotic consumption during hospitalization was obtained from pharmacy records.

### 2.2. Bacterial Reidentification, Susceptibility Testing, MIC Determination and Carbapenemase Detection

Species identification was performed using matrix-assisted laser desorption/ionization time-of-flight mass spectrometry (MALDI-TOF/MS) (Microflex; Bruker Daltonics, Bremen, Germany) as previously described [27].

Minimal inhibitory concentrations (MICs) to AMX, AMC, TIC, PIP, TZP, CF, CTX, FOX, CAZ, ceftazidime/avibactam (CAZ-AVI), cefiderocol (CEFID), ceftolozane/tazobactam (FZNTAZ), (IMP), imipenem-relebactam (IMP-RL), MP, meropenem/vaborbactam (MP-VAB), ERT, ATM, aztreonam/avibactam (ATM-VAB), AN, GM, TM, CIP, LVX, TEM, C, TIG, SXT, and colistin (COL), were determined using broth microdilution using precoated plates (Thermofisher, Les Ulis, France). MICs were interpreted according to EUCAST breakpoints as updated in 2023 [28]. Carbapenemase detection was performed using the Carba NP test as previously described [29]. The five most prevalent carbapenemase families in Enterobacterales (KPC, NDM, VIM, IMP and OXA-48-like) were searched by means of immunochromatographic assay using the NG-test Carba5 test (NG Biotech, Guipry, France) according to manufacturer’s instructions [30,31].

### 2.3. Plasmid Identification and Transfer of β-Lactam Resistance Determinants

Plasmid DNA was extracted using the Kieser method as described previously [32]. Plasmids of ca. 154, 66, 48, and 7 kb of *Escherichia coli* NCTC 50192 were used as plasmid size markers. Plasmid DNA of the isolates was analyzed by means of electrophoresis on a 0.7% agarose gel and we attempted to introduce it via electroporation into *E. coli* TOP10.

### 2.4. Whole-Genome Sequencing and Bioinformatics Analysis

Whole-genome sequencing was performed using Illumina’s Nextseq 500 on the PIBNET sequencing platform (Institut Pasteur, Paris, France). Genome assembly was performed using CLC Genomics Workbench v12 (Qiagen, Les Ulis, France). Initial genome annotation was performed using Rapid Annotation using Subsystem Technology (RAST) (https://rast.nmpdr.org/, accessed on 17 October 2023), Center for Genomic Epidemiology (CGE) services (https://www.genomicepidemiology.org/services/, accessed on 17 November 2023) [33]. The sequence type (ST) of isolates was determined according to the MLST schemes available at PubMLST (https://pubmlst.org/, accessed on 13 November 2023). Virulence gene sequences and functions were searched using virulence factors of the pathogenic bacteria database available at (http://www.mgc.ac.cn/VFs/, accessed on 15 November 2023) [28].

To assess population bias, we performed a core-genome-based phylogenetic analysis of our collection, comparing it to unrelated carbapenemase-producing ST135 *P. mirabilis* isolates from the French National Reference Center. Genome annotation was performed with Prokka v1.14.6, and pan- and core-genome identification was performed with Panaroo v1.5.1 (70% protein identity for pangenome families, 80% presence for core genes). Core genome alignment was performed using MAFFT v7.490. The best-fit model, selected via the Bayesian information criterion (BIC) in ModelFinder Plus, guided phylogenetic inference with IQ-TREE v2.0.7.

## 3. Results

### 3.1. Demographic, Clinical, and Microbiological Data

In total, 417 *P. mirabilis* isolates were recovered in Sahloul University Hospital in Sousse, Tunisia, over a 4-year period (January 2018 to December 2022), of which 262 were ampicillin-resistant (62.2%), 214 were resistant to the amoxicclin/clavulanate combination (51.3%), and 11 were resistant to all antibiotics available for clinical use in our institution (XDR*Pm*). These 11 *P. mirabilis* isolates displayed a positive CarbaNP, indicating the production of a carbapenemase. The lateral flow immunochromatographic assay NG-test Carba 5 (NG Biotech, Guipry, France) identified the production of an NDM-type carbapenemase.

These 11 *P. mirabilis* isolates were isolated from patients with several comorbidities who had experienced prolonged hospitalization in the intensive care unit (ICU), with a positive culture being yielded from various clinical specimens. Most of these patients (*n* = 10/11) were previously exposed to mechanical ventilation and intravascular devices and 6/11 had undergone surgery. Ten patients were treated with broad-spectrum antibiotics including colistin. The demographic and clinical characteristics of these patients and data concerning previous antimicrobial therapy are summarized in Table 1.

### 3.2. Antimicrobial Susceptibility Testings

All isolates were resistant to penicillins, broad-spectrum cephalosporins such as CTX (MIC > 16 mg/L), CAZ (MIC > 16 mg/L), and FEP (MIC > 16 mg/L). Carbapenem resistance was observed, with resistance to IMP and intermediate resistance to MP (MIC of IMP at 32 mg/L and MP at 4 mg/L), except for ERT (MIC ≤ 0.5 mg/L). These isolates were also resistant to the CAZ-AVI combination (MIC > 16 mg/L). All isolates except for P2-109-B1 remained susceptible to ATM and all isolates were susceptible to the ATM-AVI combination. All isolates were resistant to AN and GM (MIC > 32 mg/L), to fluoroquinolones (MIC > 2 mg/L), SXT combinations, C, tcyclines, and COL (intrinsic resistance in *P. mirabilis*).

### 3.3. Resistome and Genetic Relatedness

The draft genome of the 11 *P. mirabilis* isolates revealed an average contig number of 71.9 contigs with an L50 of 8 and an N50 of 179,556 bp. The overall GC content was of 39.1%. The average size of the genome was 4.23 Mb.

All the Illumina-sequenced *P. mirabilis* isolates carried the *bla*_NDM-1_ carbapenemase gene. One isolate (P2-109-B1) also carried the *bla*_VEB-6_ ESBL-encoding gene (Table 2). In addition, a wide variety of antibiotic resistance genes against aminoglycosides (*aph(6)-Id*, *aac(6′)-Ib*, *ant(3″)-Ia*, *aph(3′)-Ia*, *armA*, *aadA2*), fluoroquinolones (*qnrA1*, *parC* (S84I), *parE* (K84E), *gyrA* (S83I), *gyrB* (E466D)), sulfonamides (*sul2*, *sul1*), trimethoprim (*dfrA32*, *dfrA1*), macrolides (*ere(A)*, *msr(E)*), and chloramphenicol (*cat*, *floR*) (Table 2) were also observed.

**Figure 1:**
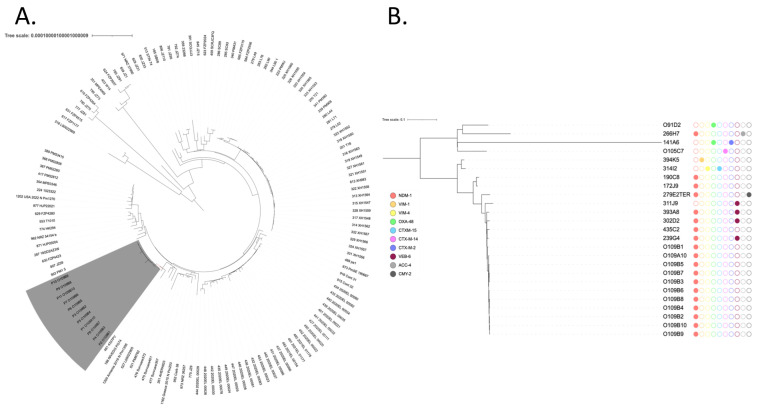
(**A**) Phylogenetic analysis of NDM-1-producing *P. mirabilis*. The eleven isolates recovered from Tunisia are colored in gray. The tree scale is indicated by the horizontal axis. (**B**) Phylogenetic tree of ST-135 *P. mirabilis* from Tunisia and from the French National Reference Center for antibiotic resistance: carbapenemase-producing Enterobacterales, Le Kremlin-Bicêtre, France. Broad-spectrum beta-lactamases are indicated by colored circles.

A global phylogenetic analysis with *P. mirabilis* genomes from GenBank revealed that the eleven *P. mirabilis* isolates were clustered, indicating a potential outbreak (Figure 1A). Accordingly, MLST analysis indicated that all of these isolates belonged to the same sequence type, ST135. To definitively decipher their clonal relationships, a second phylogenetic analysis was performed using epidemiologically unrelated carbapenemase-producing ST135 *P. mirabilis* isolates recovered from the French National Reference Center (Figure 1B). This analysis demonstrated that these eleven isolates were closely related, indicating potential cross transmission within the hospital.

This assumption is further supported by epidemiological data: all cases originated from the same ICU unit, where healthcare personnel and medical staff were shared among patients, increasing the risk of dissemination. Additionally, one affected patient, initially in the neurosurgery department, was later transferred to the POG-ICU, where the other cases were identified. These combined findings suggest a likely intra-hospital spread of the unique clone, although further epidemiological investigations would be necessary to definitively confirm direct transmission events.

### 3.4. Transfer of Carbapenem Resistance

Kieser-extracted plasmid DNA followed by transformation in *E. coli* did not allow the transfer of carbapenem resistance, likely indicating that the carbapenemase genes were carried on the chromosome. However, Kieser-extracted plasmid DNA analyzed on a 0.7% agarose gel revealed the presence of an ca. 66 kb plasmid in 9/11 isolates. No plasmid was identified in the strain harboring the *bla*_VEB-6_ ESBL gene.

## 4. Discussion

*P. mirabilis* is naturally susceptible to all beta-lactams along with aminoglycosides and fluoroquinolones [16]. However, acquired resistance to β-lactams in addition to several classes of antibiotics including SXT, fluroquinolones, fosfomycin, aminoglycosides, and sulfonamides was reported [16]. Plasmid-borne extended-spectrum β-lactamases (ESBL), AmpC β-lactamases, and carbapenemases are the most concerning β-lactamases, since they confer resistance to broad-spectrum β-lactams. We described here eleven *P. mirabilis* clinical isolates producing NDM-1 carbapenemase. Of note, in addition to NDM-1 in all isolates, one isolate (P2-109-B1) carried the minor extended-spectrum lactamase *bla*_VEB-6_, a minor variant of *bla_VEB-4_*, which confers a high level of resistance to ceftazidime, cefotaxime, and aztreonam [34]. *bla*_VEB-6_ was first reported in a strain of *P. mirabilis* recovered from a urine sample of an inpatient in Australia [35]. Interestingly, co-localization of *bla_NDM-1_* and *bla*_VEB-6_ in a same PGI (Proteus Genomic Island) has been reported in France [36]. As was observed for our 11 clinical isolates of *P. mirabilis*, the authors failed to transfer *bla*_NDM-1_ by the conjugation or electroporation in *E. coli*, despite demonstrating that the PGI was able to achieve self-excision and circularization, increasing the risk of co-transfer with another plasmid helper. This aligns with previous studies showing that integrative and conjugative elements (ICEs) and transposons play a crucial role in bacterial genome plasticity, allowing resistance determinants to persist in the chromosome while still being mobilized under specific conditions [37]. Furthermore, genomic islands with excision and integration capabilities have been implicated in the spread of antibiotic-resistant strains in healthcare settings, facilitating adaptation to selective pressures [38]. The inability to transfer *bla*_NDM-1_ in laboratory conditions does not rule out its dissemination in clinical settings, where helper elements such as conjugative plasmids or bacteriophage-mediated mechanisms may facilitate horizontal transfer, as reported in other carbapenemase-producing strains [39,40]. These findings highlight the complexity of resistance gene transmission and underscore the need for continuous surveillance of such elements in hospital environments.

While carbapenem-resistant Enterobacterales (CRE) have become a pressing threat to public health, current research in several countries has revealed that *P. mirabilis*, which produces carbapenemases, is still uncommon [41,42,43]. However, multidrug-resistant (MDR) *P. mirabilis* isolates harboring carbapenemase genes such as *bla*_OXA-48-like_, *bla*_KPC_, *bla*_NDM_, *bla*_VIM_, *bla*_IMP_, and the main CHDLs from *A. baumannii*, *bla*_OXA-23_ and *bla*_OXA-58_, are increasingly being reported [16,22,44,45]. They have frequently been described with co-resistance to fluoroquinolones, aminoglycosides, and co-trimoxazole [46,47,48].

Few cases of NDM-producing *P. mirabilis* have been described worldwide. In Tunisia, only one case reported by Kanzari et al. in 2018 [49] displaying the *bla*_NDM-1_ gene in an XDR*Pm* clinical isolate carrying plasmid mediated resistance to carbapenems (*bla*_NDM-1_), cephalosporins (*bla*_CMY-4_), aminoglycosides (*aph3*-VIa and *aph3*-Ia), and fluoroquinolones (*qnrA6*) has been reported. Since that report, no other descriptions have been published in Tunisia.

The acquisition of *bla_NDM-1_* is of special concern in relation to *P. mirabilis*, which is intrinsically resistant to tetracycline, tigecycline, and colistin. Indeed, this enzyme drastically diminishes the efficacy of almost all β-lactams (except aztreonam), including the last resort, carbapenems [23]. The extensive resistance observed in our *P. mirabilis* isolates severely limits treatment options. Notably, resistance to CAZ-AVI but susceptibility to ATM-AVI suggests that ceftazidime-avibactam combined with aztreonam may be a viable alternative against these NDM-1 producers [50].

Given the lack of effective β-lactams, cefiderocol remains a promising option due to its activity against NDM-producing pathogens [51]. These findings underscore the urgent need for antimicrobial stewardship, strict infection control, and continuous surveillance to contain the spread of multidrug-resistant *P. mirabilis*.

Beyond antimicrobial resistance, the pathogenicity of *P. mirabilis* is further enhanced by its virulence factors. The production of urease and protease contributes to stone formation and tissue necrosis, facilitating bacterial persistence in the host [8,52]. Additionally, all isolates carried *hpmA* and *hpmB*, which play a crucial role in hemolytic activity and urovirulence [53]. The presence of multiple flagellar genes (*flgN*, *flhA*, *fliA*, *fliC*, *fliF*, *fliG*, *fliL*, *fliP*) supports the bacterium’s motility and colonization abilities, despite the absence of *flaD*, which is not essential for swarming [26,54]. Key virulence factors such as *ptA*, *zapA*, *mrpA*, *pmfA*, *mrpH*, and *atfA* contribute to biofilm formation and increased antibiotic resistance, further complicating treatment [24,25,26,55]. Moreover, the utilization of phosphate transport and iron uptake systems enhances bacterial survival and pathogenic potential, emphasizing the multidimensional threat posed by these isolates [56,57].

This study reports the first outbreak of NDM-producing *P. mirabilis* in Africa, highlighting the emergence of this multidrug-resistant pathogen in Tunisia and its potential to spread within healthcare settings. Genomic analysis revealed evidence of clonal dissemination, suggesting nosocomial transmission and underscoring the urgent need for enhanced infection control measures. The resistance profile of these isolates, particularly their low MICs to ertapenem, raises concerns about the potential underestimation of carbapenem resistance in *P. mirabilis*. Further genomic and phenotypic studies are crucial, especially regarding OXA-23-producing strains with low carbapenem MICs, to better assess the clinical relevance and effectiveness of carbapenem therapy in such cases.

Beyond its clinical impact, the spread of NDM-producing *P. mirabilis* poses a serious public health threat, reinforcing the need for continuous surveillance, robust antimicrobial stewardship programs, and strict containment strategies to prevent the further dissemination of multidrug-resistant bacteria in hospital settings.

## Figures and Tables

**Table 1:** Extensively drug-resistant *Proteus mirabilis* (XDR*Pm*) isolates, including their antibiotic resistance profiles, associated invasive procedures, and patient outcomes.

	Date Isolation	Ward ^a^	Specimen ^b^	Age (Years)	Gender ^c^	Date of Hospitalization	Invasive Procedure (Site of Insertion) ^d^	Surgery	Treatment Prior to the Isolation of XDR*Pm* ^e^	Outcome ^f^
**P1-109-A10**	07.09.21	POG-ICU	Urine	51	M	21 July 21	CVC (jugular) + ***UC***	Tracheostomy–Gastrostomy	AMC + CST	Died on 27 November 2021
**P2-109-B1**	11.10.21	A-ICU	BPBS	64	M	28 August 21	CVC + AC + ***UC***	None	IMP + GM + CAZ + CIP + CST	Died on 03 November 2021
**P3-109-B2**	05.02.22	POG-ICU	BPBS	65	M	19 January 22	CVC (jugular) + AC (radial) + ***UC***	None	IMP + E + CST	Died on 07 February 2022
**P4-109-B3**	05.04.22	A-ICU	VC	64	M	05 March 22	CVC (femoral) + AC (radial) + ***UC***	Leg external fixator	IMP + CST	Discharged on 14 May 2022
**P5-109-B4**	07.04.22	POG-ICU	BPBS	51	M	24 March 22	NA	None	IMP + TEC + CST	Died on 08 April 2022
**P6-109-B5**	05.05.22	A-ICU	VC	47	F	25 November 22	CVC (femoral) + AC (radial) + ***UC***	surgical evacuation of expansive frontal hematomas	N/A	Transferred to NS on 06 November 2022
**P7-109-B6**	13.05.22	A-ICU	VC	37	M	21 April 22	CVC (jugular) + AC + ***UC***	Tracheostomy -	IMP + AN + TEC + CST	Died on 16 October 2022
**P8-109-B7**	29.05.22	*Neurosurgery*	Urine	52	F	15 March 22	CVC (jugular) + AC (radial) + ***UC***	Tracheostomy–Gastrostomy	CAZ + IMP + VAN + MTZ + CST	Discharged on 01 June 2022
**P9-109-B8**	08.08.22	A-ICU	Urine	60	F	04 July 22	CVC (jugular) + AC (radial) + ***UC***	none	TEC + CST	Died on 23 August 2022
**P10-109-B9**	27.09.22	A-ICU	BPBS	68	M	10 September 22	CVC (femoral) + AC + UC	Tracheostomy–Gastrostomy	IMP + VAN + CST	Dead on 28 September 2022
**P11-109-B10**	04.10.22	A-ICU	Blood	37	M	21 April 22	CVC (jugular) + AC (radial)	Tracheostomy–Gastrostomy	IMP + AN + TEC + CST	Died on 16 October 2022

^a^ N/A, not available; A-ICU, anesthesia-ICU; POG-ICU, post-op general anesthesia ICU. ^b^ BPBS, blind protected bronchial sampling. ^c^ M, male; F, female. ^d^ CVC (jugular venous catheter) + AC (arterial catheter) + UC (urinary catheter). ^e^ CST, colistin; CAZ, ceftazidime; GM, gentamicin; CIP, ciprofloxacin; E, erythromycin; TEC, teicoplanin; AN, amikacin; MTZ, metronidazole; VAN, vancomycin; IMP, imipenem. MICs were determined using precoated plates (Thermofischer, Les Ulis, France). MICs were interpreted using EUCAST breakpoints as updated in 2023 [28]. ^f^ NS, neurosurgery.

**Table 2:** Genomic features and epidemiological insights into multidrug-resistant *Proteus mirabilis* isolates reported in the present study: analysis of the resistome, virulome, disinfectant resistance, and sequence typing.

Isolates ID	RESISTOME				Potential Virulence Factors	ST
	Beta-Lactamases	Fluoroquinolone	Aminogycoside	Others		
**P1-109-A10**	*bla_NDM-1_*	*qnrA1, * *parC (S84I)* *gyrA(S83I)*	*aph(6)-Id, aac(6′)-Ib, ant(3″)-Ia, aph(3′)-Ia, armA, aadA1,aadA2*	*qacE sul1, sul2, dfrA1, dfrA32, ere(A), mph(E), msr(E), cat, floR, tet(C), tet(J)*	*aipA, pta, zapA, ireA, hpmA, hpmB, mrpA, pmfA, mrpH, pmpA, atfA, rcsD, ureC, ureG, flhA, FliA, fliC, fliF, fliG, fliL, fliP*	ST135
**P2-109-B1**	*bla_VEB-6_* *, bla_NDM-1_*	*qnrA1, parC (S84I) gyrA(S83I)*	*aph(6)-Id, aac(6′)-Ib, ant(3″)-Ia, aph(3′)-Ia, armA, aadA2*	*qacE sul1, sul2, dfrA1, dfrA32, ere(A), mph(E), msr(E), cat, floR, tet(A), tet(C), tet(J)*	*aipA, pta, zapA, ireA, hpmA, hpmB, mrpA, pmfA, mrpH, pmpA, atfA, rcsD, ureC, ureG, flhA, FliA, fliC, fliF, fliG, fliL, fliP*	ST135
**P3-109-B2**	*bla_TEM-2_* *,bla_NDM-1_*	*qnrA1, * *parC (S84I)* *gyrA(S83I)*	*aph(6)-Id, aac(6′)-Ib, ant(3″)-Ia, aph(3′)-Ia, armA, aadA2*	*qacE sul1, sul2, dfrA1, dfrA32, ere(A), mph(E), msr(E), cat, floR, tet(C), tet(J)*	*aipA, pta, zapA, ireA, hpmA, hpmB, mrpA, pmfA, mrpH, pmpA, atfA, rcsD, ureC, ureG, flhA, FliA, fliC, fliF, fliG, fliL, fliP*	ST135
**P4-109-B3**	*bla_TEM-2_* *,bla_NDM-1_*	*qnrA1, * *parC (S84I)* *gyrA(S83I)*	*aph(6)-Id, aac(6′)-Ib, ant(3″)-Ia, aph(3′)-Ia, armA, aadA2*	*qacE sul1, sul2, dfrA1, dfrA32, ere(A), mph(E), msr(E), cat, floR, tet(C), tet(J)*	*aipA, pta, zapA, ireA, hpmA, hpmB, mrpA, pmfA, mrpH, pmpA, atfA, rcsD, ureC, ureG, flhA, FliA, fliC, fliF, fliG, fliL, fliP*	ST135
**P5-109-B4**	*bla_TEM-2_* *,bla_NDM-1_*	*qnrA1, * *parC (S84I)* *gyrA(S83I)*	*aph(6)-Id, aac(6′)-Ib, ant(3″)-Ia, aph(3′)-Ia, armA, aadA2*	*qacE sul1, sul2, dfrA1, dfrA32, ere(A), mph(E), msr(E), cat, floR, tet(C), tet(J)*	*aipA, pta, zapA, ireA, hpmA, hpmB, mrpA, pmfA, mrpH, pmpA, atfA, rcsD, ureC, ureG, flhA, FliA, fliC, fliF, fliG, fliL, fliP*	ST135
**P6-109-B5**	*bla_TEM-2_* *,bla_NDM-1_*	*qnrA1, * *parC (S84I)* *gyrA(S83I)*	*aph(6)-Id, aac(6′)-Ib, ant(3″)-Ia, aph(3′)-Ia, armA, aadA2*	*qacE sul1, sul2, dfrA1, dfrA32, ere(A), mph(E), msr(E), cat, floR, tet(C), tet(J)*	*aipA, pta, zapA, ireA, hpmA, hpmB, mrpA, pmfA, mrpH, pmpA, atfA, rcsD, ureC, ureG, flhA, FliA, fliC, fliF, fliG, fliL, fliP*	ST135
**P7-109-B6**	*bla_TEM-2_* *,bla_NDM-1_*	*qnrA1, * *parC (S84I)* *gyrA(S83I)*	*aph(6)-Id, aac(6′)-Ib, ant(3″)-Ia, aph(3′)-Ia, armA, aadA2*	*qacE sul1, sul2, dfrA1, dfrA32, ere(A), mph(E), msr(E), cat, floR, tet(C), tet(J)*	*aipA, pta, zapA, ireA, hpmA, hpmB, mrpA, pmfA, mrpH, pmpA, atfA, rcsD, ureC, ureG, flhA, FliA, fliC, fliF, fliG, fliL, fliP*	ST135
**P8-109-B7**	*bla_TEM-2_* *,bla_NDM-1_*	*qnrA1, * *parC (S84I)* *gyrA(S83I)*	*aph(6)-Id, aac(6′)-Ib, ant(3″)-Ia, aph(3′)-Ia, armA, aadA2*	*qacE sul1, sul2, dfrA1, dfrA32, ere(A), mph(E), msr(E), cat, floR, tet(C), tet(J)*	*aipA, pta, zapA, ireA, hpmA, hpmB, mrpA, pmfA, mrpH, pmpA, atfA, rcsD, ureC, ureG, flhA, FliA, fliC, fliF, fliG, fliL, fliP*	ST135
**P9-109-B8**	*bla_TEM-2_* *,bla_NDM-1_*	*qnrA1, * *parC (S84I)* *gyrA(S83I)*	*aph(6)-Id, aac(6′)-Ib, ant(3″)-Ia, aph(3′)-Ia, armA, aadA2*	*qacE sul1, sul2, dfrA1, dfrA32, ere(A), mph(E), msr(E), cat, floR, tet(C), tet(J)*	*aipA, pta, zapA, ireA, hpmA, hpmB, mrpA, pmfA, mrpH, pmpA, atfA, rcsD, ureC, ureG, flhA, FliA, fliC, fliF, fliG, fliL, fliP*	ST135
**P10-109-B9**	*bla_TEM-2_* *,bla_NDM-1_*	*qnrA1, * *parC (S84I)* *gyrA(S83I)*	*aph(6)-Id, aac(6′)-Ib, ant(3″)-Ia, aph(3′)-Ia, armA, aadA2*	*qacE sul1, sul2, dfrA1, dfrA32, ere(A), mph(E), msr(E), cat, floR, tet(C), tet(J)*	*aipA, pta, zapA, ireA, hpmA, hpmB, mrpA, pmfA, mrpH, pmpA, atfA, rcsD, ureC, ureG, flhA, FliA, fliC, fliF, fliG, fliL, fliP*	ST135
**P11-109-B10**	*bla_TEM-2_* *,bla_NDM-1_*	*qnrA1, * *parC (S84I)* *gyrA(S83I)*	*aph(6)-Id, aac(6′)-Ib, ant(3″)-Ia, aph(3′)-Ia, armA, aadA2*	*qacE sul1, sul2, dfrA1, dfrA32, ere(A), mph(E), msr(E), cat, floR, tet(C), tet(J)*	*aipA, pta, zapA, ireA, hpmA, hpmB, mrpA, pmfA, mrpH, pmpA, atfA, rcsD, ureC, ureG, flhA, FliA, fliC, fliF, fliG, fliL, fliP*	ST135

## Data Availability

The assembled genomes of strains from the current study have been deposited in GenBank under the accession numbers JAWLIO000000000, JAWMAP000000000, JAWKAW000000000, JAXFYQ000000000, JAWMTJ000000000, JAWMAQ000000000, JAWMAN000000000, JAWONU000000000, JAXFYP000000000, JAWLVG000000000, JAWMAO000000000.
